# Molecular architecture of polycomb repressive complexes

**DOI:** 10.1042/BST20160173

**Published:** 2017-02-15

**Authors:** Emily C. Chittock, Sebastian Latwiel, Thomas C.R. Miller, Christoph W. Müller

**Affiliations:** European Molecular Biology Laboratory (EMBL), Structural and Computational Biology Unit, Meyerhofstrasse 1, 69117 Heidelberg, Germany

**Keywords:** polycomb group protein, PRC1, PRC2, PR-DUB, transcription regulation

## Abstract

The polycomb group (PcG) proteins are a large and diverse family that epigenetically repress the transcription of key developmental genes. They form three broad groups of polycomb repressive complexes (PRCs) known as PRC1, PRC2 and Polycomb Repressive DeUBiquitinase, each of which modifies and/or remodels chromatin by distinct mechanisms that are tuned by having variable compositions of core and accessory subunits. Until recently, relatively little was known about how the various PcG proteins assemble to form the PRCs; however, studies by several groups have now allowed us to start piecing together the PcG puzzle. Here, we discuss some highlights of recent PcG structures and the insights they have given us into how these complexes regulate transcription through chromatin.

## Introduction

The development of a multicellular eukaryotic organism is a highly complex process that requires exact control of specific transcriptional programs in a spatially and temporally regulated manner. To facilitate such control, the eukaryotic genome is packaged into nuclei as chromatin; a nucleoprotein complex that can be physically remodeled in response to epigenetic modifications on DNA and histone proteins, either restricting or permitting access to the DNA for all DNA-templated processes [1,2].

The polycomb group (PcG) proteins are a diverse and conserved group of proteins that function as epigenetic modifiers and transcriptional regulators. They were initially identified as transcriptional repressors of homeotic (HOX) genes in genetic screens in *Drosophila* [3–6] and are now known to be essential for embryonic development, stem cell differentiation and tissue homeostasis from flies to vertebrates [7,8]. In humans, misregulation of the PcG proteins leads to a wide range of malignancies, most notably various forms of cancer [8].

**Figure 1.**
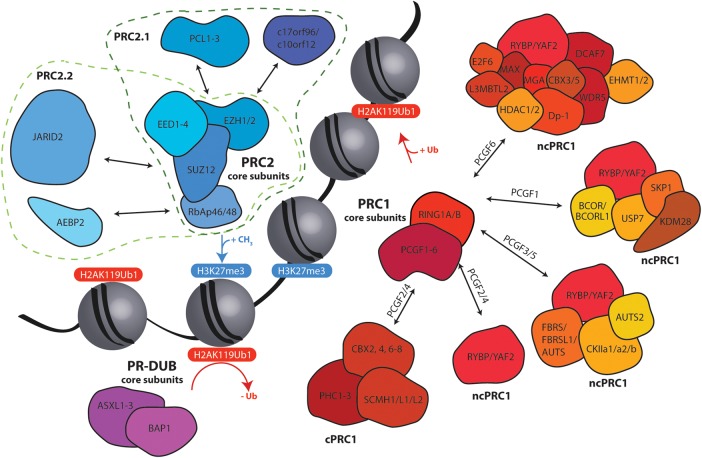
Human polycomb complexes functionally interact to regulate histone H3K27 methylation and histone H2A K119 ubiquitination. Polycomb proteins characteristically have a core enzymatic component that interacts with additional core or accessory factors to regulate enzymatic activity or genomic targeting, thus regulating the biological output of specific complexes. PRC1 complexes deposit H2AK119 ubiquitination, which stimulates PRC2–JARID2–AEBP2 activity, whereas PRC2 deposits H3K27me3, which also stimulates both PRC2 and PRC1. This produces a feedforward loop that is proposed to amplify polycomb signals over large genomic regions to mediate transcriptional repression. In contrast, PR-DUB removes H2AK119 ubiquitination deposited by PRC1 and mediates polycomb-target repression through a distinct, as-yet-unknown mechanism [9,28,29,48]. PCL, polycomblike; PHC1, polyhomeotic-like protein 1; SCMH, sex combs on midleg homologue; RYBP, RING1 and YY1 binding protein; YAF, YY1-associated factor; FBRS, probable fibrosin-1; FBRSL, FBRS-like; RING, really interesting new gene; AUTS, autism susceptibility; CKII, casein kinase II; BCOR, BCL6 (B cell lymphoma 6) co-repressor; BCORL1, BCOR-like 1; USP, ubiquitin-specific protease; SKP, S-phase kinase-associated protein; HDAC, histone deacetylase; L3MBTL, lethal (3) malignant brain tumour-like; MGA, MAX gene-associated; MAX, myc-associated factor X; WDR, WD-repeat containing; Dp-1, DRFT1 polypeptide-1; EHMT, euchromatic histone methyltransferase; E2F6, E2F (E2 factor) transcription factor 6.

PcG functional diversity derives from structural diversity and our current understanding suggests that the PcG proteins form three distinct groups of enzymatic complexes, each with the ability to carry out a specific epigenetic modification (Figure 1) [9,10]: polycomb repressive complex 1 (PRC1) complexes are E3 ubiquitin ligases that monoubiquitinate lysine 119 of histone H2A (H2AK119ub1) and also perform ubiquitination-independent chromatin compaction, and possibly interact directly with the transcription machinery; Polycomb Repressive DeUBiquitinase (PR-DUB) opposes the action of PRC1 by deubiquitinating H2AK119; polycomb repressive complex 2 (PRC2) complexes are methyltransferases that target histone H3 lysine 27 for mono-, di- and trimethylation (H3K27me1, 2, 3). Despite these well-characterized enzymatic activities, how these modifications bring about transcriptional repression and how these complexes functionally interact with one another is still largely unknown.

While our knowledge of the biological importance of the PcG family and the modifications these complexes perform has been expanding, obtaining detailed structural information of these complexes had proved challenging, severely limiting our understanding of their regulation and the targeting to their biological substrate — nucleosomes. Recently, however, several new structures of PcG components and subcomplexes [11–15], or homologous complexes [16,17], have shed light on how the proteins of this family function together. In this mini-review, we discuss recent advances in our understanding of the molecular architecture of the mammalian PRCs, and discuss how key findings from these structures inform our understanding of their regulation and their ability to modify chromatin.

## Polycomb repressive complex 1

**Figure 2.**
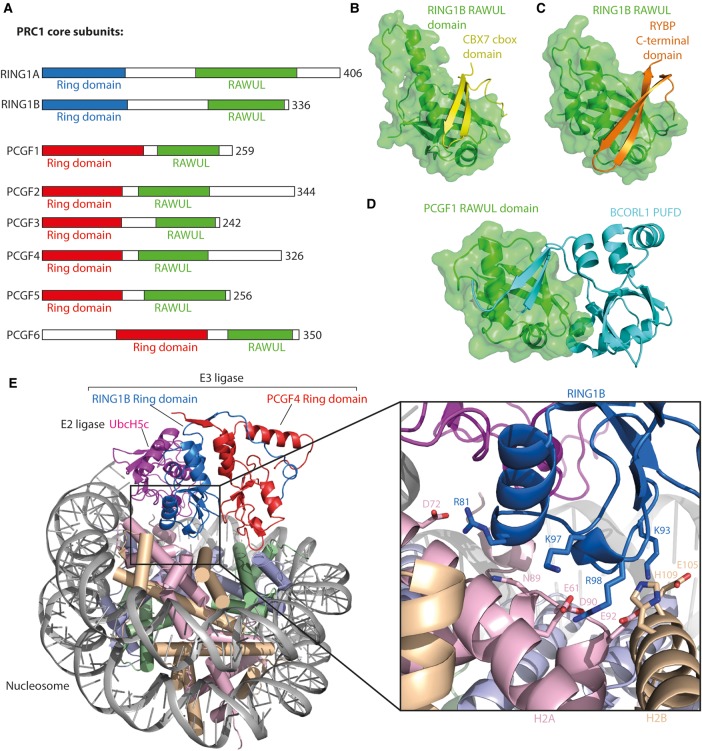
The PRC1. (**A**) Domain architecture of the PRC1 core proteins: the RING1 proteins and the PCGF proteins. (**B**) Structure of the RING1B RAWUL domain/CBX7 cbox domain (PDB: 3GS2, [27]). (**C**) Structure of the RING1B RAWUL domain/RYBP C-terminal domain (PDB: 3IXS, [27]). (**D**) Structure of the PCGF1 RAWUL domain/BCORL1 PFUD domain (PDB: 4HPL, [30]). (**E**) Structure of the RING1B ring domain–UBcH5c fusion/PCGF4/nucleosome complex (PDB: 4R8P, [11]). Single E2–E3 ligase shown for clarity.

The PRC1 complexes are a diverse family of multicomponent complexes that contribute to polycomb repression by monoubiquitination of H2AK119 (H2AK118 in *Drosophila*), non-enzymatic compaction of chromatin and possibly by direct interaction with the transcription machinery [18–22]. PRC1 complexes contain a core consisting of a RING1 protein (RING1A or RING1B) and a polycomb group ring finger protein (PCGF1–6; Figure 2A), which heterodimerize via their N-terminal ring domains [23]. The RING1/PCGF heterodimer forms a scaffold for PRC1 assembly, with both proteins interacting with additional PRC1 components via their C-terminal RAWUL (Ring finger And WD40 Ubiquitin-Like) domains [24,25]. RING1 RAWUL domains interact with a Chromobox protein (CBX 2, 4, 6–8) or RYBP (or its homolog YAF2) in a mutually exclusive manner (Figure 2B,C) [25–28], while each PCGF protein seems to selectively interact with specific binding partners, defining the makeup of the PRC1 complexes (Figure 1), and therefore their level of ubiquitination activity, as well as their genomic localization [28,29]. Historically, mammalian PRC1 complexes have been classified as canonical PRC1 (cPRC1), so-called because its subunit composition is homologous to that of the *Drosophila* PRC1 complex, or non-canoncial PRC1 (ncPRC), which describes all other PRC1 complexes; classification based on the PCGF protein identity has recently been suggested as a more useful alternative [28], reviewed by Aranda and colleagues [9].

There are several crystal structures available for RING1 and PCGF RAWUL domains interacting with their binding partners [27,30]. In the structure of the RING1B RAWUL domain/CBX7 cbox domain complex (Figure 2B), RING1B RAWUL comprises an extensive β-sheet region with a long central helix. CBX7 cbox also forms a β-sheet, which makes an intermolecular β-sheet with RING1B, and a C-terminal loop that packs against the RING1B β-sheet. The structure of the RING1B RAWUL domain/RYBP C-terminal domain complex (Figure 2C) shows RYBP interacting with RING1B in a very similar way to CBX7, despite significant sequence differences. Minimal RING1B RAWUL-binding partners are 30–35 residues in length [27], whereas PCGF RAWUL domains seem to require larger surfaces for binding and interact more selectively with their binding partners [30]. In *in vitro* assays, the RAWUL domains of PCGF1 and PCGF3 bind the BCORL1 PUFD (PCGF ubiquitin-like fold discriminator), whereas the RAWUL domains of PCGF2 and PCGF4 bind PHC1. The structure of the PCGF1 RAWUL/BCORL1 PUFD complex (Figure 2D) also shows the two components forming an intermolecular β-sheet, although the strands for the BCORL1 β-sheet come from the N- and C-termini, rather than from a contiguous region as is seen for CBX7 and RYBP [30]. In a recently solved crystal structure of the PCGF1/BCORL1/KDM2B/SKP1 complex, the PCGF1 RAWUL/BCORL1 PUFD interface straddles the KDM2B F-box leucine-rich repeat, with both PCGF1 and BCORL1 contributing key residues for binding. The PCGF3/BCORL1 complex, however, was unable to associate with KDM2B [31]. These structural data fit with proteomic studies by Gao et al. [28], showing that the identity of the PCGF subunit of PRC1 largely determines the overall subunit composition of the PRC1 complex (except that complexes containing PCGF2 (Mel18) or PCGF4 (Bmi1) may contain either RYBP/YAF2 or a CBX protein). Similarly, recent proteomic studies by Kloet et al. [29] show a switch in the predominant PCGF protein bound to Ring1B during mouse embryonic stem cell (ESC) differentiation, with PCGF6 occupying 60% of Ring1B in ESCs but PCGF4 occupying 54% of Ring1B in differentiating neural progenitor cells. This switch is accompanied by a corresponding change in the abundances of PCGF6- and PCGF4-specific accessory subunits. Structures of further PCGF RAWUL domains with their binding partners are required to understand the mechanism of PCGF binding selectivity.

The ubiquitination activity of PRC1 complexes is performed by the Ring domain of the RING1 protein, which functions as an E3 ligase, transferring ubiquitin from an E2 ligase to the H2AK119 target [19,32,33]. Some insights into the mechanism and targeting of PRC1 E3 ligase activity toward H2AK119 have been provided by a recent crystal structure of the RING1B/PCGF4 Ring domain heterodimer fused to the E2 ligase UbcH5c (forming the E2–E3 complex) bound to a nucleosome (Figure 2E) [11]. The RING1B Ring domain has an N-terminal extension, which wraps around the PCGF4 Ring domain, guided by the helix-forming C-terminal extension of the PCGF Ring domain. The N-terminal loop of the RING1 proteins is essential for this unusual mode of Ring domain dimerization, which leads to a substantial enhancement of RING1 activity against nucleosomes [32,33]. Previously solved structures of the RING1B Ring domain/PCGF4 Ring domain complex [32,33] and the E2–E3 complex alone [34] are very similar to those of the nucleosome-bound E2–E3 complex, showing that no major structural changes occur upon nucleosome binding.

Targeting of PRC1 complexes to nucleosomes occurs via several distinct mechanisms (Figure 2E). First, the basic residues of RING1B, which were previously shown to be required for *in vitro* nucleosome ubiquitination and DNA binding [34], interact with the nucleosome's acidic patch, explaining *in vitro* and *in vivo* data suggesting that the nucleosome acidic patch is required for H2A ubiquitination [35]. Specifically, arginines and lysines from RING1B insert into an acidic pocket on H2A, with Glu105 and His109 of H2B creating a ridge adjacent to the pocket and forming van der Waal's contacts with the aliphatic part of the RING1B Arg98 side chain. This ‘arginine-anchor’ mode of binding to the nucleosome acidic patch is observed in all crystal structures of chromatin factors bound to nucleosomes to date, although the proteins have no shared structural features beyond an arginine-rich sequence [36–40]. RING1B R81 also interacts with the acidic patch via a second site not previously observed in chromatin factor/nucleosome structures. Additionally, PCGF4 interacts with the H3 and H4 histone folds, and UbcH5c makes contacts with DNA at the nucleosomal dyad. These extensive interactions between the E2–E3 complex and the nucleosome precisely position the complex to specifically ubiquitinate H2A K119 (and to a lesser extent K118).

From a structural perspective, the PRC1 E3 ligase activity is currently better understood than PRC1 chromatin compaction and interaction with the transcription machinery; however, the relative importance of the various mechanisms and how they bring about transcriptional repression is still unclear. Interestingly, biological data suggest that these two mechanisms are functionally distinct. Disruption of the E3 ligase activity of *Drosophila* PRC1 Ring protein Sce shows that H2A ubiquitination is dispensable for the repression of canonical PcG target genes, but is nonetheless required for viability [41]. Similarly, the E3 ligase of Ring1B is not essential for the early stages of mouse development. Complete knockout of Ring1B leaves mice unable to complete gastrulation (embryonic day 6.5–7) [42], while endogenous expression of catalytically inactive Ring1B leads to relatively minor morphological defects and lethality only after 15.5 days [43]. Experiments in mouse ESCs have shown that E3 ligase activity of PRC1 is not required for its localization or for chromatin compaction at HOX genes, but is essential for efficient repression of PRC1 target genes [44].

The idea that these modes of chromatin modification by PRC1 complexes are distinct is supported by the fact that not all PRC1 complexes appear to be important for ubiquitination *in vivo* [45]; this is despite the structural similarity between different PRC1 E2–E3 structures (e.g. PCGF4 vs. PCGF5 E2–E3 complexes) [34,46], and despite all six PCGF/RING1B complexes showing intrinsic catalytic activity that depends on binding to the H2A acidic patch in a broadly similar manner [46]. This may be due to certain PCGF proteins being less able to stimulate RING1 E3 ligase activity, as was observed for PCGF2 *in vitro* (in spite of being able to form a stable Ring domain heterodimer) [19], or being unable to correctly position the E2–E3 complex for H2AK119 ubiquitination (mutation of basic residues in the PCGF4 nucleosome-binding surface to the corresponding PCGF5 residues greatly decreases E3 ligase activity of the PCGF–RING1B complexes [46]). Alternatively, the presence of certain additional PRC1 subunits, or the non-enzymatic compaction of chromatin, may sterically hinder PRC1 interaction with H2AK119 or the E2 ligase, precluding the ubiquitination of chromatin. Further structures are needed to decipher how the diverse subunit compositions of the PRC1 complexes determine their functional outputs by tuning their genomic targeting, and both their enzymatic and non-enzymatic activities to regulate transcription in such a highly co-ordinated way *in vivo*.

## Polycomb repressive complex 2

The PRC2 complexes are *S*-adenosyl-l-methionine (SAM)-dependent histone methyltransferases (HMTases) that catalyze the mono-, di- and trimethylation of H3K27. Originally identified as a repressor of HOX genes in *Drosophila* [4,47], mammalian PRC2 has recently been classified as two distinct complexes (PRC2.1 and PRC2.2; Figure 1A; [48]), which together have been implicated in a range of biological processes including cell proliferation, stem cell plasticity, carcinogenesis and X inactivation (reviewed in refs [49–51]). Both human PRC2 complexes share a catalytic subunit, EZ homolog 2 (EZH2), which contains an enzymatic SET (Su(var)3-9, enhancer of zeste (EZ) and Trithorax) domain belonging to the EZ family of HMTases [52,53]. *Ezh2* knockout results in embryonic lethality in mice [54], highlighting the fundamental role of EZH2 in development. EZH2 is highly expressed in embryonic and proliferating cells and shows a strong catalytic preference toward trimethylation of H3K27 [55]. The mutually exclusive homolog, EZH1, however, shows reduced HMTase activity, and its role remains controversial.

**Figure 3.**
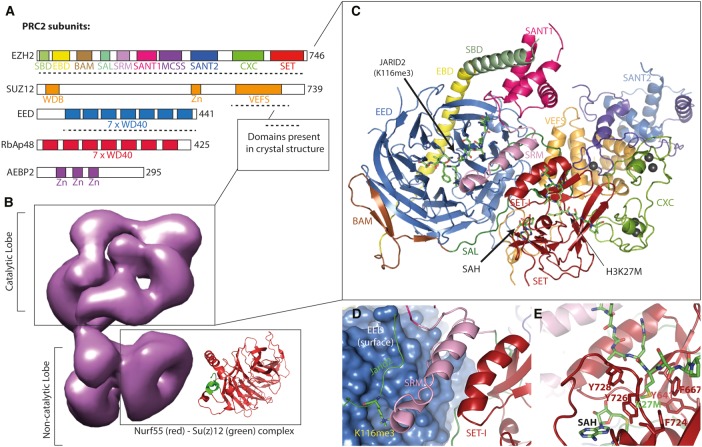
The PRC2. (**A**) Domain architecture of PRC2 subunits found in the negative stain electron microscopy (EM) reconstruction of PRC2 (**B**): EZ homolog 2 (EZH2), EED, SUZ12, retinoblastoma-binding protein p48 (RbAp48), adipocyte enhancer-binding protein 2 (AEBP2). Dashed lines indicate domains present in the recent human X-ray crystal structure of the PRC2 core complex (**C**). (**B**) Human PRC2 complex solved using negative stain EM at 21 Å resolution (EMDB: 2236, [13]). The X-ray crystal structure of the *Drosophila* Nurf55 (red) and Su(z)12 (green) complex (PDB: 2YB8, [66]) is shown alongside EM density from the noncatalytic lobe, which is proposed to contain the homologous human RbAp48 WD40 domain. (**C**) Structure of the human PRC2 core complex comprising EZH2, EED and the VEFS domain of SUZ12 in complex with JARID2 stimulatory peptide, the cofactor SAH and the inhibitory H3K27M peptide (PDB: 5HYN, [15]). (**D**) The stimulatory peptide, JARID2 K116me3, binds to EED (blue, surface representation) causing the nearby SRM (pink) to adopt an α-helical conformation, which in turn abuts SET-I, further stabilizing the active site and stimulating its activity. (**E**) H3K27M inhibitory peptide (green) bound in the substrate recognition channel of the SET domain (red). The SAH cofactor is also displayed in green below the substrate recognition pocket.

A low-resolution electron microscopy structure of the human PRC2.2 complex, comprising EZH2, suppressor of Zeste 12 homolog (SUZ12) and embryonic ectoderm development (EED) in combination with the accessory subunits AEBP2 and RbAp48 (but lacking JARID2), revealed a two-lobed conformation (Figure 3A,B) [13]. The catalytic lobe consists of EZH2, EED, the C-terminus of SUZ12 and AEBP2, whereas RbAp48 and the N-terminus of SUZ12 form the noncatalytic lobe. Interestingly, unlike homologous SAM-dependent SET domains, such as G9a and DIM-5, which are active without additional subunits [56,57], EZH2 alone is catalytically inactive, requiring EED (one of the four isoforms) and SUZ12 to form a minimal, active PRC2 complex [58]. This is because the isolated human EZH2 SET domain adopts an autoinhibitory conformation, where the SAM cofactor-binding pocket and substrate entry channel are incomplete [59,60].

Recent crystal structures of minimal PRC2 core complexes showed that EZH2 possesses many domains that act as interaction platforms for EED and SUZ12 VEFS, facilitating the formation of the active enzyme (Figure 3C) [12,14,15]. The N-terminal region of EZH2 forms a tight strap around EED, reinforced by the interaction of SANT1 with the SANT1-binding domain. The EZH2 SET activation loop interacts with EED and extends outward toward the back of the SET domain. This interaction is mediated by a conserved stretch of acidic residues in the N-terminal region of the VEFS box and results in a 20° anticlockwise movement of the SET-I helix, away from the substrate entry channel and toward the cofactor-binding pocket. This movement simultaneously completes the cofactor-binding pocket and unblocks the substrate entry channel, resulting in catalytic activation [12,14].

The complex of EZH2, SUZ12 VEFS and EED activates the SET domain; however, further catalytic stimulation is achieved upon the binding of its product, H3K27me3, and is thought to be important for the propagation of the repressive mark. H3K27me3 is recognized by an aromatic cage on the WD40 domain of EED [61]. The stimulatory recognition motif (SRM) of EZH2 binds to K3K27me3 (already bound by EED), causing the SRM to adopt an α-helical structure that abuts and stabilizes the SET-I, stimulating the SET domain [14]. PRC2 also trimethylates K116 of JARID2, a PRC2 accessory subunit, which can also be bound by EED, mimicking the stimulatory effects of H3K27me3 (Figure 3E). The modification of JARID2 and subsequent autostimulation of PRC2 activity may be important in regulating PRC2 recruitment and activity in a chromatin landscape devoid of H3K27me3, explaining its vital role early in development [62].

The structures of the minimal PRC2 complex were also capable of shedding light on how an oncogenic point mutation (H3K27M) in a single histone H3 allele can bind and inhibit PRC2, leading to a global reduction in H3K27me3 and the development of pediatric glioblastomas [63,64]. Although an initial structure of the *Chaetomium thermophilum* minimal PRC2 complex suggested an alternative mode of H3K27M binding in the EZH2 active site [14], it is now clear that this mutated methionine actually binds in the same position as the lysine in wild-type histone H3 [15]. The entry site of this pocket is composed of largely aromatic residues and this creates a hydrophobic environment that appears to preferentially accommodate the methionine, rather than lysine side chain, thus enabling the K27M histone tail to bind to PRC2 with a higher affinity and inhibit it from methylating wild-type histone H3 (Figure 3E) [15,64].

The *Drosophila* histone-binding protein Nurf55, a WD40 repeat protein homologous to the interchangeable RbAp46/RbAp48 subunits in mammals, binds to the WD40-binding domain in the N-terminus of Su(z)12 (SUZ12 in mammals) and is positioned in the noncatalytic lobe of PRC2 (Figure 3B) [13,65,66]. Nurf55 anchors PRC2 to the N-terminus of H3 and is sensitive to trimethylation of H3K4 (a mark of actively transcribed chromatin), which reduces the affinity of Nurf55 for H3. Interestingly, H3K4me3 was also found to reduce the HMTase activity of PRC2, but without impairing binding of PRC2 to nucleosomes [66]. This allosteric down-regulation requires the VEFS domain of SUZ12, occurring only when H3K4me3 is in *cis*, on the same histone tail as the H3K27 substrate.

H3K36me2/3, another mark of actively transcribed chromatin, also reduces the HTMase activity of PRC2 [66], but this appears to be dependent on which subtype of PRC2 encounters the mark. The inhibitory effect of H3K36me3 on the PRC2 core is abolished in the presence of PHF1 (characteristic of PRC2.1), which can bind H3K36me3 via its conserved Tudor domain [67]. This suggests that PRC2–PHF1 complexes may be important for the spread of the H3K27me3 mark into H3K36me3-rich regions [67]. Structural validation and characterization of these PRC2 complexes in the presence of H3K4me3 and K36me2/3 peptides (or nucleosomes) are required to fully understand the precise allosteric mechanisms that regulate PRC2 in regions of actively transcribed chromatin.

The recent structural data discussed above have gone a long way toward describing the activation and stimulation of the minimal PRC2 complex; however, the precise mechanisms of PRC2 recruitment to target loci in mammals remain contentious. In *Drosophila*, this process is better understood and seems to center on polycomb response elements (PREs) and the PhoRC complex [68–76]. However, the role of PREs in mammalian polycomb recruitment appears less pronounced as only a few have been identified [77] and other mechanisms therefore appear to be important. In both *Drosophila* and humans, PRC2-mediated H3K27me3 targets PRC1 E3 ligase activity to H2AK118/119 and monoubiquitination of H2AK118/119 recruits PRC2 complexes that contain AEBP2 and JARID2, creating a positive feedback loop and further stimulating H3K27me3 catalysis and subsequent PRC1 recruitment [71,78–80]. Although this feedback loop explains a proportion of PRC2 recruitment in mammals, it should be noted that PRC1 distribution does not overlap entirely with PRC2 and H3K27me3, and depletion of H3K27me3 does not reduce H2AK119ub1 levels in mouse ESCs [81]. This suggests that the processes occurring in mammals are much more complex and currently believed to involve a combination of histone modifications, coding and noncoding RNAs, and additional interaction partners. Uncovering the structural mechanisms that allow mammalian PRC2 to be targeted to specific genomic locations by integrating these diverse signals will be a major ongoing area of PRC2 research.

## Polycomb Repressive DeUBiquitinase

The PR-DUB complex is a histone deubiquitinase that removes the H2AK119ub1 mark deposited by PRC1. PR-DUB is a relatively recent addition to the PRCs; the gene encoding the catalytic subunit Calypso was first identified as a PcG gene in 2007 in *Drosophila* genetic screens [6], and the PR-DUB complex of Calypso with its only known binding partner, ASX (additional sex combs), was first described in 2010 [10]. Calypso belongs to the ubiquitin C-terminal hydrolase (UCH) class of deubiquitinases and requires the presence of ASX for activity against H2AK118ub1 nucleosomes *in vitro* [10]. Deletion of PR-DUB leads to an almost 10-fold increase in bulk H2AK118ub1 levels and derepression of the HOX genes in *Drosophila* [6,10]. This was a somewhat unexpected phenotype, given that PR-DUB deubiquitinase activity antagonizes the H2AK118 ubiquitination activity of PRC1, which is also required for HOX gene repression. It could be that PR-DUB activity is required to release ubiquitin, or H2AK118ub1-binding factors that become sequestered elsewhere in the genome and thereby limit H2AK118 ubiquitination by PRC1 [82].

**Figure 4.**
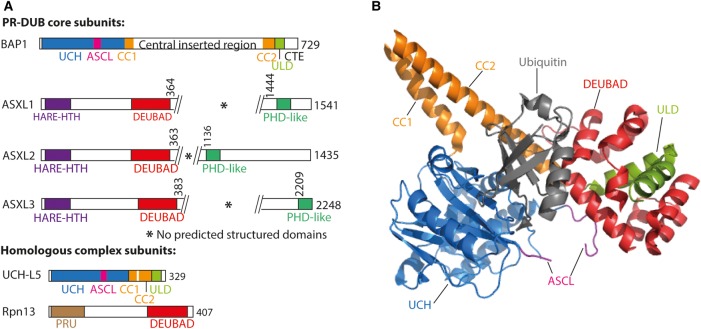
The PR-DUB complex. (**A**) Domain architecture of PR-DUB core subunits BAP1 and ASXL proteins, as well as the UCH-L5 and Rpn13 proteins, which form a homologous deubiquitinase complex. UCH: ubiquitin carboxyl hydrolase; ASCL: active site crossover loop; CC1: coiled-coil N-terminal strand; CC2: coiled-coil C-terminal strand; ULD: Uch37-like domain; CTE: C-terminal extension; HARE-HTH: HB1, ASXL1, Restriction Endonuclease Helix-Turn-Helix; DEUBAD: DEUbiquitinase Adaptor; PHD-like: atypical PHD finger; PRU: Plecktrin-like Receptor for Ubiquitin. (**B**) Structure of the UCH-L5/Rpn13/ubiquitin complex (PDB: 4UEL, [16]).

The mammalian Calypso homolog BAP1 (BRCA1-associated protein 1) also interacts with ASX homologs (ASXL1–3; Figure 4A), and, like Calypso in *Drosophila*, BAP1 is inactive against H2AK119ub1 nucleosomes in the absence of an ASXL protein [10]. However, BAP1 can also form complexes with many other proteins, including the tumor suppressor BRCA1, host cell factor-1 (HCF-1), *N*-acetylglucosamine transferase, the forkhead box transcription factors FOXK1/2, MBD family proteins MBD5/6, transcription factor YY1 and ubiquitin-conjugating enzyme UBE20 [48,83–87]. Several of these binding partners are implicated in non-PcG BAP1 functions, such as DNA double-strand break repair, DNA replication and cell cycle progression, and are themselves substrates for BAP1 deubiquitinase activity [88–90].

The interaction between BAP1 and the ASXL proteins is mediated by the ∼120-residue DEUBAD (DEUBiquitinase ADaptor) domain of the ASXL proteins [91], and this region is sufficient for activation of BAP1 in *in vitro* H2AK119ub1 nucleosome deubiquitination assays [92]. There is currently no published structural information for the PR-DUB complex; however, there are several crystal structures available for the closely related UCH protein UCH-L5 (UCH 37), including a structure with the Rpn13 DEUBAD domain [16,17,93,94]. BAP1 and UCH-L5 both have an N-terminal UCH catalytic domain (46% identical) and share a UCH 37-like domain (ULD) at the C-terminus (36% identical; Figure 4B). A coiled coil separates these two domains in both UCH-L5 and BAP1; however, BAP1 has an additional ∼350 amino acid insertion between the two strands. UCH-L5 and BAP1 both interact with the DEUBAD domains of their binding partners via their ULDs. UCH-L5 forms a 1:1 complex with Rpn13 and INO80G DEUBADs; in contrast, experimental data suggest a 2:1 ratio for the BAP1/ASXL1 complex [29,83,92].

Activation of UCH-L5 by Rpn13 DEUBAD is mediated by allosteric effects that give the complex a greater affinity for ubiquitin than UCH-L5 alone. The active site crossover loop (ASCL), a feature shared by all UCH family members, is thought to restrict substrate access to the active site based on substrate size [95]. Interestingly, this loop is disordered in the crystal structure of UCH-L5 alone [93], but in the structures of UCH-L5 with Rpn13 DEUBAD, some residues of the ASCL are observed to interact with Rpn13, directing the ASCL away from the active site (Figure 4B). Rpn13 DEUBAD binding also positions the ULD in a favorable conformation, relative to the UCH domain, for ubiquitin binding [16,17]. Mutagenesis experiments suggest that these mechanisms are conserved for BAP1 activation by ASXL1 DEUBAD. The conserved ‘NEF’ region of ASXL1 DEUBAD is important in BAP1 activation; the corresponding region in UCH-L5 stabilizes bound ubiquitin [92].

Key features of the BAP1/ASXL1 complex that cannot be modeled based on the UCH-L5/DEUBAD structures are the C-terminal extension (CTE) of BAP1 (significantly longer than the UCH-L5 CTE, which is absent from the UCH-L5 crystal structures) and the additional inserted region between the strands of the coiled coil (Figure 4A). The CTE is a ∼20-residue arginine- and lysine-rich extension after the BAP1 ULD, which is predicted to be disordered. The CTE is required for BAP1 activity against H2AK119ub1 nucleosomes, but not for general deubiquitinase activity against the model substrate ubiquitin-AMC. The cationic nature of the CTE was shown to be important for interaction with the nucleosome (both H2AK119ub1 and wild type) *in vitro*, although, somewhat surprisingly, experimental data suggest that neither the nucleosome acidic patch, nor the nucleosomal DNA, mediates this interaction [92].

The central region of BAP1 is predicted to be disordered. Sites within this central region have been identified as important for binding non-ASXL complex components; for example, the HCF-binding motif and the phosphorylated threonine residue that mediates BAP1's interaction with FOXK2 have been mapped to this region [96,97]. Additionally, phosphorylation of six sites within this region has been shown to be important for BAP1's role in DNA double-strand break repair [88]. Since this region is not conserved in *Drosophila* Calypso, it may be required for the alternative, non-PcG functions of BAP1.

While BAP1 and ASXL1 DEUBAD alone are sufficient for nucleosome deubiquitination *in vitro*, additional domains or proteins may be involved in targeting the complex *in vivo.* Structural predictions and phylogenetic analyses identify three globular regions of ASXL1: the DEUBAD domain, an N-terminal HARE-HTH (HB1 ASXL1 Restriction Endonuclease Helix-Turn-Helix) domain and a C-terminal atypical PHD finger [91,98]. HTH domains and PHD fingers are known to bind DNA and methylated lysines within histones, respectively, and hence may be involved in PR-DUB nucleosome binding, though this has not been investigated experimentally. Additional complex components, including HCF-1, FOXK2 and YY1, have all been implicated in recruiting BAP1 or PR-DUB to specific genetic loci [86,96,99]. With the information currently available, it is difficult to define a human PR-DUB complex beyond the core components BAP1 and ASXL1–3; however, it is likely that BAP1 will form part of a diverse range of complexes, only some of which perform PcG functions.

## Concluding remarks

Mammals have evolved a large complement of PcG proteins that could potentially combine to assemble hundreds of PRCs, each with distinct, overlapping or redundant functional roles in transcription regulation. Recent structural advances have meant that we are now beginning to develop a picture of the core components of these complexes and their primary enzymatic mechanisms, yet much remains to be discovered. While there appears to be a set of stable core subunits for each PRC, the accessory subunits of the complexes are highly dynamic [29]. We still know little about the mechanistic and functional variability introduced to these complexes by accessory factors, including how these factors affect enzymatic activity, PRC recruitment to chromatin and the spreading of the epigenetic marks deposited by the PcG proteins. We know even less about how this variability defines each unique complex's role in organismal development, stem cell maintenance and differentiation, and cancer. The field of structural biology of polycomb proteins is therefore still somewhat in its infancy, with many fundamental questions in the field remaining to be answered. Given the diversity of polycomb complexes, their inherent size and flexibility (as well as that of their constituent PcG proteins) and the typically transient nature of the interactions between the core and accessory components with each other, with histones and with DNA, expanding our structural knowledge is a significant challenge. Fortunately, recent developments in cryo-electron microscopy may facilitate our moves toward this goal, by allowing us to investigate complexes that would otherwise be intractable by X-ray crystallography or NMR.

## Abbreviations

AEBP: adipocyte enhancer binding protein
ASCL: active site crossover loop
ASX: additional sex combs
ASXL: ASX homologs
BAP1: BRCA1-associated protein 1
CBX: Chromobox
CTE: C-terminal extension
DEUBAD: DEUBiquitinase ADaptor
EED: embryonic ectoderm development
ESC: embryonic stem cell
EZ: enhancer of zeste
EZH2: EZ homolog 2
HARE-HTH: HB1 ASXL1 Restriction Endonuclease Helix-Turn-Helix
HCF-1: host cell factor-1
HMTases: histone methyltransferases
HOX: homeotic
JARID: jumonji- and AT-rich interaction domain
KDM2B: lysine-specific demethylase 2B
PcG: polycomb group
PCGF1–6: polycomb group ring finger proteins
PHD: plant homeodomain
PHF: PHD finger protein
PRCs: polycomb repressive complexes
PR-DUB: Polycomb Repressive DeUBiquitinase
PREs: polycomb response elements
PUFD: PCGF ubiquitin-like fold discriminator
RAWUL: Ring finger and WD40 Ubiquitin-Like
RbAp48: retinoblastoma binding protein p48
SAM: *S*-adenosyl-l-methionine
SANT: Swi3 (switching-defective protein 3) Ada2 (adaptor 2) N-CoR (nuclear receptor co-repressor) TFIIIB (transcription factor IIIB)
SRM: stimulatory recognition motif
SUZ12: Su(z)12
UBE: ubiquitin-conjugating enzyme
UCH: ubiquitin C-terminal hydrolase
ULD: UCH 37-like domain
VEFS: VRN2-EMF2-FIS2-SUZ12.
